# Efficacy of Levofloxacin Loaded Nonionic Surfactant Vesicles (Niosomes) in a Model of *Pseudomonas aeruginosa* Infected Sprague Dawley Rats

**DOI:** 10.1155/2020/8815969

**Published:** 2020-10-27

**Authors:** Satish Jankie, Jenelle Johnson, Amusa Sarafadeen Adebayo, Gopal Krishna Pillai, Lexley Maureen Pinto Pereira

**Affiliations:** ^1^School of Pharmacy, University of the West Indies, St. Augustine, Trinidad and Tobago; ^2^School of Veterinary Medicine, University of the West Indies, St. Augustine, Trinidad and Tobago; ^3^College of Pharmacy, Department of Pharmaceutical Sciences, Sullivan University, Louisville, KY 40205, USA; ^4^Department of Paraclinical Sciences, University of the West Indies, St. Augustine, Trinidad and Tobago

## Abstract

This study examined the effectiveness of niosomes loaded with levofloxacin in treating *Pseudomonas aeruginosa* (American Type Culture Collection—ATCC 27853) infections in Sprague Dawley rats since these infections are becoming more common and resistant to treatment. Levofloxacin entrapped in niosomes was prepared using the thin-film hydration method and was assessed for in vitro release and stability. Three groups of six (6) animals were infected with a lethal dose of *Pseudomonas aeruginosa* via the intraperitoneal (Ip) route. At six (6) hours postinfection, the animals were treated with either drug-free niosomes (control), free levofloxacin (conventional), or levofloxacin trapped in niosomes (Ip) at a dose of 7.5 mg/kg/once daily. Blood was collected via tail snips on days 0, 1, 3, 5, 7, and 10 for complete blood counts and viable bacterial counts (CFU/*μ*l). At day 10, the animals were sacrificed, and the kidney, liver, and spleen were harvested for bacterial counts. The niosomes showed a sustained drug release profile and were most stable at 4°C. All animals in the control group succumbed to the infection; one animal from the conventional group died, and all niosome treated animals survived at day 10. The mean lymphocyte count (×10^9^) was lower for the niosome (7.258 ± 1.773) versus conventional group (17.684 ± 10.008) (*p* < 0.03) at day ten (10). Neutrophil counts (×10^9^) were lower for the niosome (2.563 ± 1.609) versus conventional (6.2 ± 6.548) (*p* < 0.02) groups. Though CFUs in the bloodstream were comparable for both treatment groups, the niosome treated group showed a significant reduction of CFUs in the liver, kidney, and spleen versus the conventional group (1.33 ± 2.074) vs (5.8 ± 3.74) (*p* < 0.043), (1.5 ± 2.35) vs (9.6 ± 8.65) (*p* < 0.038) and (3.8 4.71) vs (25.6 14.66) (*p* < 0.007), respectively. These findings indicate that niosome is promising as a drug delivery system in treating systemic infections, but further work using niosomes with surface modification is recommended.

## 1. Introduction

Bacterial resistance is on the rise partly due to the increased usage, overuse, and inappropriate use of antimicrobial agents. *Pseudomonas aeruginosa* is an opportunistic pathogen responsible for a significant quantity of nosocomial infections worldwide [1]. Treating this organism is problematic because of its intrinsic pathogenic nature and ability to develop resistance [2–5]. Current therapeutic options in treating pseudomonal infections are limited to beta-lactams, aminoglycosides, and fluoroquinolones [1], with the latter being the only oral anti-pseudomonal option. Because of this ease of availability and administration, it is widely used in clinical practice, and this has been linked to a sharp rise in antimicrobial resistance to fluoroquinolones [6, 7].

Therapeutic failure in treating infectious diseases occurs as a result of the drug's inability to accumulate at the infection site, sub-therapeutic drug levels in the blood or organs, and inactivation/degradation of the drug within the body compartments [8, 9]. The search for new agents with improved intracellular efficacy to counter the increase in antimicrobial resistance is not promising, as new agents being developed are derivatives of established antibiotic classes, which face the problem of cross-resistance [10]. There is an urgent need to develop antibacterial agents that are effective against gram-negative organisms.

Increased antibacterial resistance and the lack of new drug approvals prompt the use of an alternative approach utilizing a carrier system to encapsulate drugs that enhance delivery to target the site of infection. Nonionic surfactant vesicles (niosomes) can improve the action of existing pharmaceutical agents by targeting their release at the site of desired action so that widespread distribution is minimized. In vitro work conducted with niosomal levofloxacin showed a reduction in minimum inhibitory concentrations (MIC) of ciprofloxacin-resistant *Pseudomonas aeruginosa* [11]. The reduction in MIC was obtained with niosomes without surface modification; we therefore used this formulation in this baseline in vivo study. Although a lot of work focused on liposomes [12], niosomes have numerous advantages in their procurement and preparation. The raw materials are cheaper, and production does not require highly specialized equipment or strictly controlled experimental conditions. This makes niosomes a convenient drug delivery system for investigating the efficacy of antimicrobial agents in systemic infections.

*P. aeruginosa* can avoid the innate immune response and proliferate in the bloodstream before spreading to various organs by a mechanism that is not fully understood [8]. The use of a drug carrier system is significant since obligate or facultative intracellular organisms can conceal, survive, and increase their numbers inside the phagocytic cells of the reticuloendothelial system. It is thought that their location within these cells shields them from the hosts' immune response and antibacterial agents [9, 13]. Niosomes can act as an agent of targeted drug delivery and have a positive impact on drug sensitivity, as the use of this technology can allow higher levels of the drug in the cytoplasm of microbes. This study investigates the effect of the niosomes containing levofloxacin on drug delivery, animal survival, hematological, and microbiological parameters when infected with a lethal dose of *Pseudomonas aeruginosa*.

## 2. Methodology

### 2.1. Niosomes Preparation and Characterization

Niosomes were prepared by the thin-film hydration method using cholesterol, sorbitan monostearate, and dicetylphosphate (Sigma Aldrich, Canada) in the molar ratio of 47.5 : 47.5 : 5 [14]. Chloroform was solvent utilized as all the components were readily soluble, together with the advantage of its low boiling point and high evaporation rate. Due to the solubility of the compounds used, the ease of evaporation, and the nature of thin-film formation, no additional solvents were utilized. Sorbitan monostearate (span 60) was the nonionic surfactant utilized in this study, and dicetylphosphate was used to induce a negative charge on the niosome surface, which would result in mutual repulsion between niosomes to prevent fusion. Cholesterol was used in the preparation of niosomes as it increases membrane stability, decreases the fluidity of the membrane, and also alters membrane permeability [15].

The entrapment efficiency of levofloxacin in niosomes was optimized by investigating the volume of rehydration, speed of rehydration, the temperature of rehydration, and time of rehydration as reported previously [11]. Levofloxacin (>98% pure, Sigma Aldrich, Canada) (257 mg) was dissolved in 6 mls of chloroform by gentle agitation in a 500 ml round bottom flask. Cholesterol (269 mg), sorbitan monostearate (299 mg), and dicetylphosphate (40 mg) were added to the flask and slowly dissolved by gentle agitation. Once completely dissolved, ten (10) grams of glass bead (3-4 mm diameter) was then added to the contents of the round bottom flask.

### 2.2. Dehydration of Organic Solvent

The round bottom flask containing the organic solvent mixture was attached to a rotary flash evaporator (Buchi Rotavapor® R-215). The speed of rotation was set to 40 rpm, and the round bottom flask was lowered into the water bath, which was maintained at 40°C. The pressure was set to 40 psi, and the organic solvent was evaporated under reduced pressure for 30 minutes. A thin and uniform film was deposited on the inner wall of the flask and the surfaces of the glass beads. Traces of the organic solvent were removed by allowing a stream of nitrogen to flow through the evaporator for 5 minutes, which also prevented oxidation of the thin film [16]. When the organic solvent was completely removed, the film was allowed to dry for 30 minutes.

### 2.3. Rehydration of the Thin Film

To the film, 4 mls of phosphate-buffered saline (PBS- pH 7.4) was added and rehydrated for 4 hours at 40°C. The resulting solution was removed using a pipette and placed in 1 ml plastic Eppendorf tubes. The glass beads were rinsed with 1 ml PBS, and the resulting solution was collected. This process was repeated three times.

### 2.4. Determining the Quantity of Encapsulated Drug

The tubes containing the niosome suspension were centrifuged at 20,000 rpm for one hour at 4°C. After centrifugation, the supernatant was removed, and the contents of the vial were resuspended in PBS and centrifuged again under the same conditions. This process was repeated three times. The supernatant was used for UV-VIS Spectrophotometry at 288 nm to determine the quantity of the unencapsulated levofloxacin.

(1) $$\begin{array}{c} \%\text{ of drug retention}=\frac{\left(\text{Co}-\text{Cf}\right)100}{\text{Co}}, \end{array}$$

where Co denotes the amount of fluoroquinolone initially used and Cf represents the unencapsulated drug in the supernatant.

### 2.5. In Vitro Release Study

In vitro release properties were determined by the dialysis tube method, and particle size analysis was conducted using the Mastersizer 2000 as reported previously [11]. A beaker containing 500 mls of PBS (pH 7.4) was placed on a hot-magnetic stirring plate. The temperature was maintained at 37 ± 2°C, and the magnetic stirrer was set at 100 rpm. A dialysis tube was soaked in distilled water for 5 minutes to soften its ends. One end was tied, whilst the other end was left open. Five grams of levofloxacin niosome drug suspension was placed in the tube. The niosomal suspension was completely immersed in the PBS solution (pH 7.4). One milliliter samples were drawn from PBS at time intervals- 15 min, 30 min, 1 hour, 2, 4, 6, 8, 12, 18, and 24 hours. Once a sample was removed, the volume was replaced to maintain the 500 mls on the beaker. Samples collected were analyzed by UV-VIS spectrophotometry, a wavelength of 282 nm, to determine the quantity of levofloxacin that diffused out of the dialysis tube into the surrounding medium.

### 2.6. Stability Study

Three (3) batches of niosomal loaded levofloxacin (20 mls) were prepared at refrigerated temperature (4 ± 2°C), room temperature (25 ± 2°C) and incubated (37 ± 2°C). One (1) ml samples were then retrieved at weekly intervals from each batch. The quantity of drug in the supernatant was determined by UV-VIS spectroscopy. The amount of drug that leaked out of niosomes at each week over eight weeks was then calculated.

### 2.7. Animal Experimentation

Eighteen female Sprague Dawley rats (200–250 g) were divided into three groups and individually housed in metabolic cages. They were allowed seven days to acclimatize to the local environment before any experimental work was commenced. Animals were infected with a lethal dose *of Pseudomonas aeruginosa* (ATCC 27853) via the intraperitoneal route as determined previously [17]. Treatment commenced at six hours postinfection and was continued for five days. The first (control) group was treated with drug-free niosomes, the second group with niosome encapsulated levofloxacin at a dose of 7.5 mg/kg/dose every 24 hours, whilst the third group received conventional nonencapsulated levofloxacin at the same dose.

Blood was obtained via tail bleeds on days 0, 1, 3, 5, 7, and 10. The white blood cell count was determined manually using a hemocytometer at 10X magnification with reduced light. Bacterial counts in blood were determined by placing a sterile 1 mcl loop into the blood sample and streaking onto blood agar plates (BAP) using the standard microbiological technique. The plates were incubated at 37°C for 18–24 hours, after which colony counts were performed. For *P. aeruginosa*, the confirmatory test using the OXIDASE reagent was performed.

At day 10 postinfection, the animals were sacrificed, and organs were collected for colony counts. Bacterial counts in organs were determined by making an incision into the median lobe of the liver, the middle of the spleen, and the left kidney using a sterile surgical blade. A 1 *μ*l loop was inserted into each organ, and the sample was streaked onto BAP, which was incubated at 37°C for 18 hours after which colony counts were performed.

### 2.8. Ethical Approval

The study protocol was approved by the Department of Graduate Studies, University of the West Indies, St. Augustine. The application for animal research was approved by the Animal Ethics Committee, Faculty of Medical Sciences, University of the West Indies. The research conducted using Sprague Dawley rats followed the Principles of Laboratory Animal Care.

### 2.9. Statistical Analysis

Statistical analysis to determine the intergroup variation was carried out using the Student's *t*-test or one-way analysis of variance (ANOVA) for independent samples using Minitab 16 statistical software. The value was considered to be significant if *p* < 0.05.

## 3. Results

**Figure 1:**
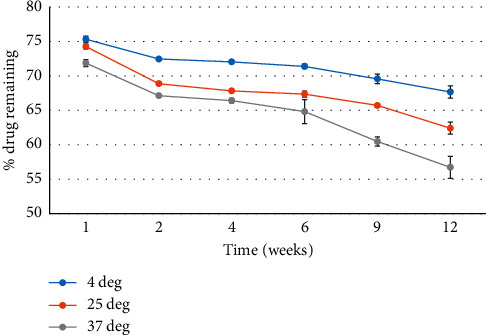
Percentage of levofloxacin remaining within niosomes over eight weeks when stored at 4, 25 and 37°C (*p* < 0.036).

The entrapment of levofloxacin in niosomes was optimized, and average encapsulation (34.23 ± 1.86%) was obtained. The eight-week drug release stability study of levofloxacin in niosomes indicated temperature-dependent stability. Leakage of levofloxacin from niosomes was slowest at 4°C when compared with 25°C and 37°C. At 8 weeks, 67.7% levofloxacin remained entrapped in niosomes that were stored at 4°C. The release of the drug fitted best into a first-order kinetic model (correlation coefficient = 0.9909) (Figure 1).

**Figure 2:**
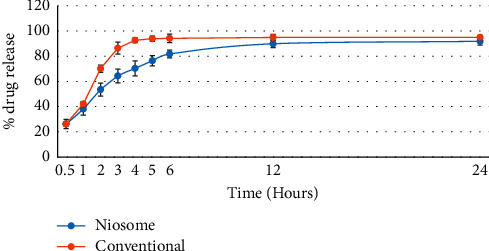
In vitro release of niosome and conventional levofloxacin formulations (*p* < 0.33).

**Figure 3:**
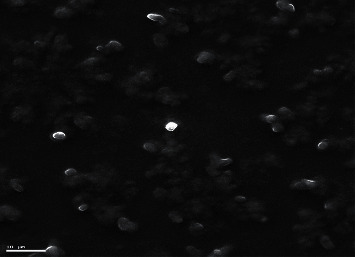
Scanning electron microscopy image of levofloxacin niosomes (×1310).

**Figure 4:**
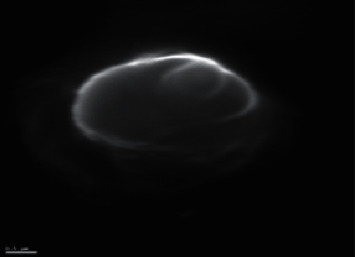
Scanning electron microscopy image of levofloxacin niosomes (×20,800).

The dissolution profiles of drugs from levofloxacin niosomes mimicked a sustained-release preparation with drug released in 6 hours (81.8% ± 2.9) and at 24 hours (91.9% ± 2.9). Data from the dissolution test were fitted to different kinetic models, and the first-order kinetic model appears to be the most suitable for describing the drug release of levofloxacin from niosomes as determined previously [11] (Figure 2). The average particle size of levofloxacin niosomes ranged between 8 and 15 *μ*m [11]. Figures 3 and 4 represent scanning electron microscopy images of the niosomes in the samples.

**Figure 5:**
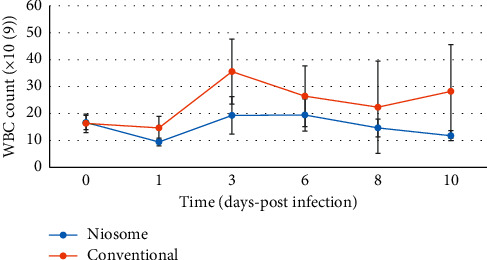
Graph showing White blood cell count in Sprague Dawley rats after infection with *Pseudomonas aeruginosa* and treatment with niosome and conventional levofloxacin (*p* < 0.036).

**Figure 6:**
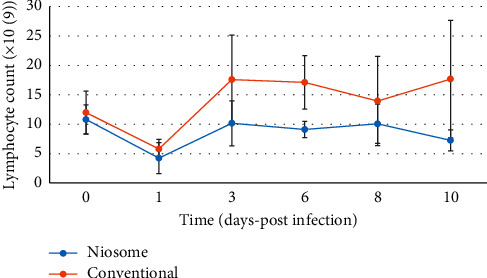
Graph showing lymphocyte counts in Sprague Dawley rats after infection with *Pseudomonas aeruginosa* and treatment with niosome and conventional levofloxacin (*p* < 0.03).

**Figure 7:**
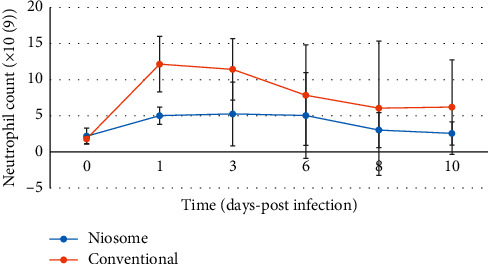
Graph showing neutrophil counts in Sprague Dawley rats after infection with *Pseudomonas aeruginosa* and treatment with niosome and conventional levofloxacin (*p* < 0.049).

All animals in the control group (treated with PBS) died within 8–12 hours after the infective organism was administered. One animal treated in the conventional levofloxacin group died, whilst all the niosome treated animals survived the infection on day ten. Before the bacterial challenge, there were comparable baseline counts for all hematological parameters tested. White blood cells (WBC), lymphocyte, and neutrophil count all showed a familiar pattern after infection and treatment had commenced (Figures 5–7), respectively.

**Figure 8:**
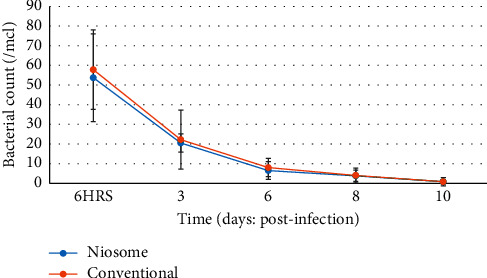
Graph showing bacterial counts in Sprague Dawley rats after infection with *Pseudomonas aeruginosa* and treatment with niosome and conventional levofloxacin (*p*<0.919).

At twenty-four hours postinfection, there was a decrease in WBC and lymphocyte counts. These became elevated at day three and decreased steadily till the end of the experimental period, although the conventional group showed a slight increase at day 10 compared to day 8. The same pattern was observed for lymphocyte counts during the experimental period. The conventional drug formulation showed a neutrophil count, which was higher throughout the course of the study when compared to the niosome group. The bacterial count in the bloodstream was comparable for both groups as shown in Figure 8.

The niosome group showed consistently lower colony counts in organs after infection with *Pseudomonas aeruginosa* (Table 1). The bacterial counts for the niosome group were significantly lower than those of the conventional group for the kidneys, liver, and spleen.

## 4. Discussion

The in vitro release patterns of the niosome formulation suggests a sustained release profile that can enhance drug delivery. The animals treated with the niosomal formulation showed better survival rates, reduced insult to white blood cells, and decreased bacterial count in the liver, kidney, and spleen at post mortem.

All animals in the control group succumbed to the infection suggesting the dose of *P. aeruginosa* administered was lethal and sufficient to induce death in an untreated animal. One animal in the group treated with the conventional formulation succumbed to the infection at day 3 postinfection. On post mortem, there were little changes in gross necropsy to determine a cause of death. This can be attributed to individual variation with the group, as they were all of similar age and weight and were infected with the same dose of *P. aeruginosa*.

The niosome treated animals also showed a less significant increase in the white blood cell, lymphocyte, and neutrophil counts postinfection; and it was effective in reducing bacterial counts in the liver, kidney, and spleen of the infected animals to a greater extent than the conventional drug. This study builds upon previous work conducted in our laboratory, which included niosome preparation and characterization, in vitro release patterns, and the in vitro activity against ciprofloxacin-resistant bacterial strains [11]. The in vitro studies showed a reduction in minimum inhibitory concentrations for the organisms tested, which included *P. aeruginosa* and justified the use of the formulation in an animal model. This is the first of such works in our laboratory using niosomes, and we therefore conducted the study using niosomes without surface modifications.

Bacterial infections occur when the immune response of the host is inadequate to counter the attack of invading microorganisms. The pattern observed for white blood cell count was characteristic of a left shift as seen with bacterial infections [18–20]. In our study, lymphocyte counts showed a similar “left shift” pattern, while neutrophils showed an initial decrease before rising, as these cells were recruited in the acute phase of the infection to combat the pathogen. This is a characteristic pattern observed in the acute phase response to a bacterial infection.

The white blood cell counts were higher for the group treated with the conventional formulation as opposed to the niosomal entrapped drug. Since the dose given was identical, the formulation must be responsible for less pronounced response in the niosomal group, as the colloidal carrier aids in targeting the infection [21], which reduces the impact on the body's defense mechanism. The insult to white blood cells, lymphocyte, and neutrophil counts was reduced as the drug is delivered preferentially to the site of infection, which can explain the faster return to baseline values of hematological parameters. Niosomes act similar to liposomes as carriers for antimicrobial drugs and can increase drug levels at the infection site, increase bactericidal efficacy, and enhance drug uptake whilst reducing potential adverse effects. The efficacy and impact of formulation change have been documented with liposomes [21–25]. This can be attributed to the ability of the formulation to protect the drug from degradation in the external environment and deliver it, possibly through the macrophages of the reticuloendothelial system to the areas where the bacterial pathogen resides.

The bacterial count in the bloodstream was comparable for both groups, and this could have occurred since an ATCC strain was used, which was sensitive to levofloxacin. The concentration of drug attained in the bloodstream was sufficient to successfully eradicate the organism. The difference in formulation did impact the colony counts in the liver, kidney, and spleen, which was significantly lower for the niosome treated group. When infection occurs with *P. aeruginosa*; the organism is distributed throughout the body but concentrates on the lung, liver, kidney, and spleen of the infected animal [26]. *P. aeruginosa* is an extracellular pathogen and avoids phagocytosis, which in turn facilitates extracellular multiplication, but there is evidence to suggest that it may also have an intramacrophage phase where the organism can multiply whilst being shielded from host defense mechanisms [27].

Facultative and obligate intracellular infections can resist the killing action of macrophages after active uptake. These organisms resist fusion of the lysosome with the phagosome, resist lysosomal enzymes, and may evade the effects of the phagosome and enter into the cytoplasm of the infected cell [26]. For the drug to be effective, it must concentrate within the infected cell be able to access the intracellular compartment where the organisms reside in significant concentrations before exerting its action on the target cells. In the case of gram-negative *P. aeruginosa*, the niosomes may be intact when delivered to the highly perfused organs. The gram-negative organisms lack the thick network of a peptidoglycan layer and may fuse with the niosomes, which allow for enhanced delivery of the drug.

Once this is achieved, enhanced action can occur as the drug is targeted to the areas of infection. This can explain why the bacterial counts in the niosome treated *P. aeruginosa* group were significantly lower than the counts with the conventional unentrapped drug. The niosomal formulation appears to concentrate at these organs and was able to reduce viable bacterial counts more efficiently than the conventional unentrapped drug. These results are similar to various studies done with a liposomal drug delivery system [28–30].

Limitations to the study include the use of the intraperitoneal route for inducing infection and subsequent treatment. The intravenous route was not feasible due to the large volumes of the infective dose and niosomal formulation used in the study, which was not compatible with the small animals used. Further studies using resistant organisms are warranted to determine the effect of the drug delivery system on survival, hematology, and colony-forming units in organs when a drug-resistant strain is the offending pathogen. Additional work is also required using niosomes with surface modifications such as macrophage activators, to assess niosomes as a viable drug delivery system for systemic infections.

## 5. Conclusion

Levofloxacin was encapsulated into niosomes using the think film rehydration method and showed a sustained release profile when tested in an in vitro model of drug delivery. Animal model experimentation showed that the niosomes were well tolerated by the animals as there was no sign of irritation or local reaction at the injection site. The niosome formulation showed improved results compared to the conventional drug in terms of survival, hematological, and microbiological parameters. The reduced insult to white blood cells and neutrophils suggests increased efficiency of drug delivery and action with the niosome formulation. Its positive effect on reducing bacterial counts suggest enhanced delivery to the organs where the organisms accumulate. Although these initial results of niosomes without surface modification were encouraging, further study on this formulation for antimicrobial drug delivery is recommended to establish it as an efficient candidate for treating intracellular and extracellular infections.

## Figures and Tables

**Table 1:** Bacterial counts in liver, spleen, and kidneys (1 *μ*l) on day 10, after treatment with levofloxacin (niosome) and levofloxacin (conventional) in rats inoculated with *Pseudomonas aeruginosa*.

	Niosome CFU/1 *μ*l ± SD	Conventional CFU/1 *μ*l ± SD
Liver	1.3 ± 2.074	5.8 ± 3.74^*∗*^
Kidney	1.5 ± 2.35	9.6 ± 8.65^*∗*^
Spleen	3.8 ± 4.71	25.6 ± 14.66^*∗*^

^*∗*^ *p* < 0.05 using one-way ANOVA (MINITAB 16)

## Data Availability

All data are within the article.
